# Environment and body-brain interplay affect inhibition and decision-making

**DOI:** 10.1038/s41598-022-08280-3

**Published:** 2022-03-11

**Authors:** Pierre Bouny, Marion Trousselard, Sandrine Jacob, François Vialatte, Charles Verdonk

**Affiliations:** 1 Department of Neurosciences and Cognitive Sciences, Unit of Neurophysiology of Stress, French Armed Forces Biomedical Research Institute, 91220 Brétigny-sur-Orge, France; 2 French Military Health Service Academy, 75005 Paris, France; 3 University of Lorraine, APEMAC-EPSAM EA 4360, 57006 Metz, France; 4 ESPCI Paris – PSL University, 75005 Paris, France

**Keywords:** Neuroscience, Cognitive neuroscience

## Abstract

The fine-tuned interplay of brain and body underlies human ability to cope with changes in the internal and external milieus. Previous research showed that cardiac interoceptive changes (e.g., cardiac phase) affect cognitive functions, notably inhibition that is a key element for adaptive behaviour. Here we investigated the influence on cognition of vestibular signal, which provides the brain with sensory information about body position and movement. We used a centrifuge-based design to disrupt vestibular signal in healthy human volunteers while their inhibition and decision-making functions were assessed with the stop-signal paradigm. Participants performed the standard and a novel, sensorial version of the stop-signal task to determine whether disrupted vestibular signal influences cognition as a function of its relevance to the context. First, we showed that disrupted vestibular signal was associated with a larger variability of longest inhibition latencies, meaning that participants were even slower to inhibit in the trials where they had the most difficulty inhibiting. Second, we revealed that processing of bodily information, as required in the sensorial stop-signal task, also led to a larger variability of longest inhibition latencies, which was all the more important when vestibular signal was disrupted. Lastly, we found that such a degraded response inhibition performance was due in part to the acceleration of decision-making process, meaning that participants made a decision more quickly even when strength of sensory evidence was reduced. Taken together, these novel findings provide direct evidence that vestibular signal affects the cognitive functions of inhibition and decision-making.

## Introduction

We act upon the environment while our brain continuously integrates information that comes from within and outside the body. Through the body, the brain receives information about the state of external environment, as well the body in relation to space and movement (exteroception) and the body’s internal state (interoception). The fine-tuned interplay of brain and body underlies our ability to cope with changes in the internal and external milieu, and response inhibition is a key cognitive process to adjust our behaviour accordingly.

The very few human studies that investigated how bodily signals influence inhibition focused on interoception. Rae et al. reported that cardiac cycle influences response inhibition, which has been shown to be enhanced at systole and attenuated at diastole^1^. Neuroimaging studies suggest that some of the brain areas involved in interoceptive signal processing and inhibition, notably the anterior insula and the anterior cingulate cortex, could overlap^2,3^. Interestingly, another neural system involved in response inhibition, the parietal cortex, is implicated as substrate for processing exteroceptive information and its central integration with interoceptive signals^4^. Specifically, the parietal cortex encompasses the pre-supplementary motor area and the vestibular cortex that are involved in inhibition and processing of vestibular information, respectively^2,5,6^. This implies that inhibition function might be affected by vestibular signal as a rapid, unconscious, cue to guide behaviour.

In the present study, we were interested in the potential influence of disrupted vestibular signal on response inhibition. Based on the aforementioned lines of evidence, we predicted that disruption of vestibular signal could degrade response inhibition performance. To test this hypothesis, we rotated participants in a centrifuge while the position of their vestibular system was varied radially in relation to the rotation axis in order to disrupt vestibular signal through short-term gravitational alteration. While rotating, response inhibition was assessed with the well-established stop-signal paradigm that included the classical stop-signal task^7,8^ and a modified, “sensorial” stop-signal task. Specifically, the Go subtask of the sensorial stop-signal task required participants to process bodily information for positioning a stimulus with respect to their body position (see section *Method* for detailed description of the sensorial stop-signal task). This task was especially designed for the present study to test whether disrupted exteroceptive signal influences response inhibition as a function of the relevance of exteroceptive information to the context.

Inhibition performance in the stop-signal paradigm is formalized as a race between two independent processes, a Go and a Stop process. This so-called horse-race model assumes that if the Stop process finishes first, the response is successfully inhibited; otherwise the response is erroneously executed^7^. Yet, it seems reasonable to assume that the response inhibition is determined by other aspects of the decision process as well: for example, a fast Go process must be accompanied by a fast Stop process in order to obtain an acceptable level of inhibition success^9^. Therefore, the present work also investigated the decision-making process related to the Go subtask using a well-established computational model of decision-making, that is, the diffusion decision model (DDM)^10^. Thus, by investigating both inhibition and decision-making latent components, we have the potential to provide a sophisticated understanding of how exteroceptive signal influences the different cognitive processes that are involved in the stop-signal paradigm.

Here, we show three main findings: (1) disrupted vestibular signal affects inhibition in increasing variability of longest inhibition latencies at the individual level, depending on the relevance of vestibular information to the context; (2) processing bodily information degrades response inhibition performance, particularly when bodily signal is disrupted; and (3) impaired inhibition is due in part to the acceleration of decision-making process, which involves a less conservative decision threshold and a reduced non-decisional time that ultimately lead to a poor cognitive performance.

## Materials and methods

### Participants

Fifty-nine healthy right-handed participants took part in the study. All participants reported no history of neurological or vestibular disorders, and normal or corrected-to-normal vision. Five participants did not complete all experimental conditions due to motion sickness symptoms. After the experimental session, datasets related to 16 participants were discarded because they met any of the following criteria that have been recommended for reliable estimate of inhibition latency, also known as the stop signal reaction time (SSRT)^11^: (i) percent inhibition (P_Inhib_) on Stop-trials less than 25% or greater than 75%; (ii) percent Go-omission (here named *Go failure*, GF) greater than 40%; and (iii) SSRT standard estimate (see below for its detailed computation) that is negative or less than 75 ms (ms) (see Supplementary Fig. 1). Data from the remaining 38 participants (mean age: 37 years old, SD: 10; 19 females—50%) were analyzed. Informed consent for both study participation and publication of identifying information/images in an online open access publication was obtained from each participant. All subjects were paid for their participation in the study. All procedures were approved by the Ile de France XI independent ethics committee.

### Experimental design

**Figure 1:**
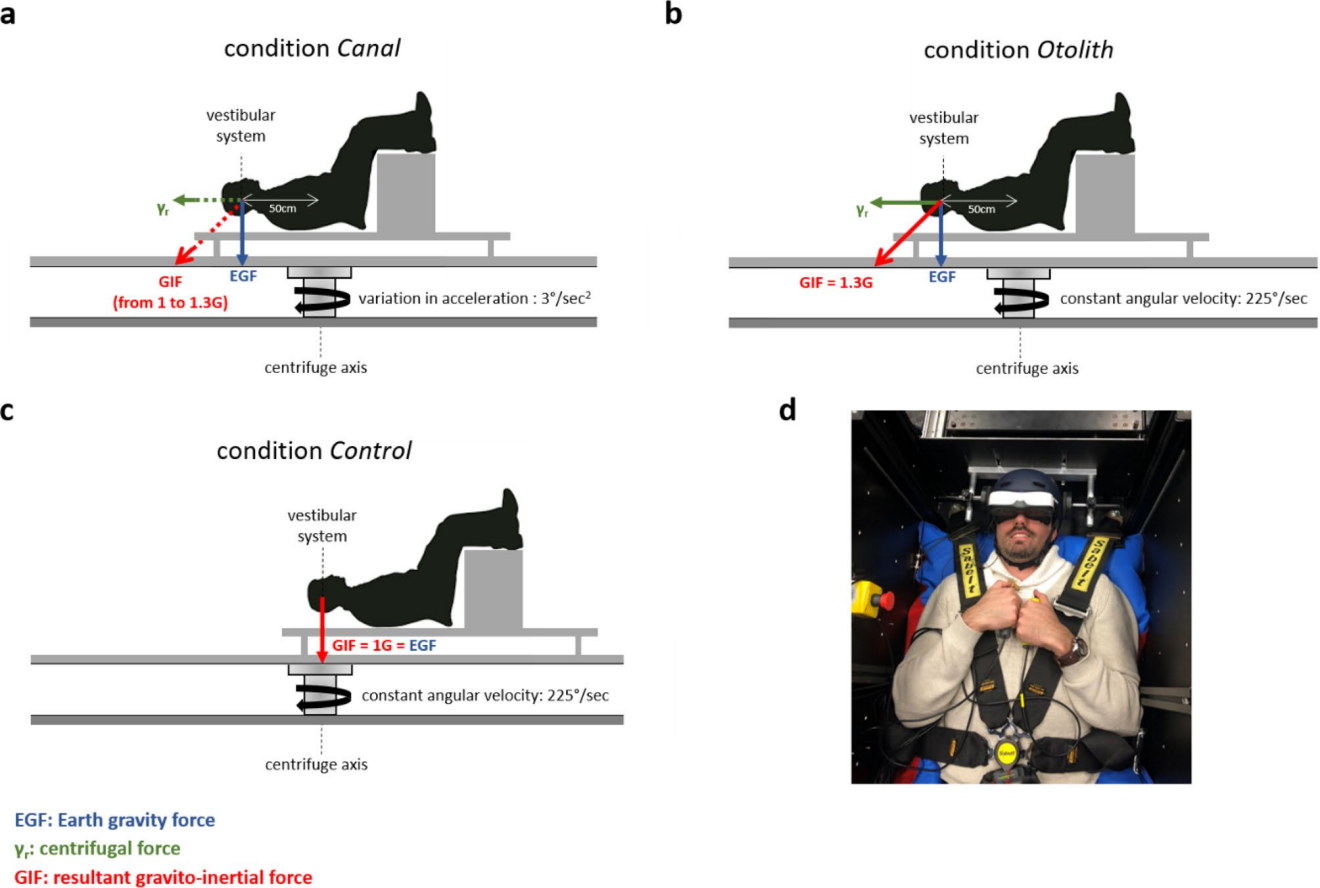
Centrifuge-based design. The resultant gravitato-inertial force (GIF) applied on the subject’s vestibular system is the vectorial sum of the Earth gravity force (EGF) and the centrifugal force. (**a–c**) The RGI was greater (conditions *Canal* and *Otolith*) or equal (condition *Control*) to EGF. In the condition *Canal*, semicircular canals were activated with centrifuge acceleration (variation in acceleration: 3°/seconds^2^). In the condition *Otolith*, only otoliths were stimulated after two minutes of rotation at constant velocity (225°/seconds), which allowed semicircular canals to return to resting activity levels. (**d**) Picture of a participant that shows the helmet-mounted display where the stimuli were presented, the key presses for response (one held on each hand), and how the head and body were fixed on the rotating platform.

Before the real recordings, participants conducted two training sessions: a first round with the motionless centrifuge to familiarize the subject with the two tasks (100 trials for each task; see below for detailed description of tasks), and a second round to familiarize the subject with rotating centrifuge. Then, participants performed the two tasks successively under three experimental conditions. The order of conditions, tasks, and mappings of the response (to the Go stimulus) were counterbalanced across participants. The three conditions were defined by the resultant gravito-inertial force (GIF) applied to the vestibular system (1 or 1.3 G) and by the vestibular organ that was stimulated (semicircular canals or otoliths). We applied gravitational alteration (1.3 G) by positioning the subject’s vestibular system 50 cm from the centrifuge axis. The condition *Canal* stimulated the semicircular canals by exposing vestibular system to a variation in acceleration (3°/second^2^) that was constant throughout centrifuge acceleration. Of note, the parameter that we experimentally controlled was the variation in acceleration of the centrifuge, and not the rotation velocity itself (which progressively increased from 0 to 225°/second in 75 s). The condition *Otolith* applied 1.3G on the otoliths by rotating centrifuge at constant angular velocity (225°/second). Of note, constant angular velocity corresponds to a variation in acceleration equal to zero, and thus prevents any potential activation of semicircular canals during rotation^12^. The condition *Otolith* was designed to start after two minutes of rotation at constant velocity to avoid any residual activation of semicircular canals that might result from the initial acceleration of centrifuge. The condition *Control* did not apply any gravitational alteration on the vestibular system since the latter was centered on the centrifuge axis (Fig. 1).

In the centrifuge, participants were lying on their back with legs flexed above the body. The subject’s body was tightly fixated using a seat belt and adjustable shoulder and hip supports, and the head aligned with the body by means of a padded helmet firmly fixated. Participants wore a helmet-mounted display (BIGEYES H1, Visionhmd, Taiwan) where the stimuli were presented, and held two key presses to provide response (Fig. 1d).

### Stop-signal tasks

In the stop-signal paradigm, participants perform a choice response time (RT) task that refers to as the Go subtask, in which they respond to a stimulus, that is, the Go stimulus. In a minority of trials (30%), the Go stimulus is followed by a Stop signal that instructs participants to withhold their response^7,8^. In the present work, the two stop-signal tasks (classical and sensorial) were implemented using Matlab 2018b (The Mathworks) and the PsychToolbox^13^. Both tasks consisted of 100 trials in the condition *Control* and 50 trials in conditions *Canal* and *Otolith* (where rotation time was limited to 75 s due to technical specifications of the centrifuge), including 70% Go- and 30% Stop-trials. The Go- and Stop-trials were presented in a pseudo-randomized and unpredictable manner to the subject. Each trial began with the presentation of a fixation dot that was replaced by the Go stimulus after 250 ms. In Stop-trials only, the Go stimulus was followed after a variable delay (the stop-signal delay, SSD) by the Stop signal, i.e. change of the Go stimulus color from white to blue, which instructed participants to withhold their response. Initially set to 200 ms, the SSD was continuously adjusted over trials with the staircase procedure to obtain a P_inhib_ around 50%^14^: after the response was successfully stopped in a Stop-trial (i.e., button press was inhibited) the SSD was increased by 75 ms, whereas when the subject did not stop successfully the SSD was decreased by 75 ms. The Go stimulus was presented until the participant responded, with a maximum response time of 1200 ms. The intertrial interval was set to 250 ms (see Supplementary Fig. 2 for the display sequence for the stop-signal tasks).

As mentioned in the introduction, the two stop-signal tasks differed by the nature of the Go subtask, which allowed us to test whether disrupted vestibular signal influences response inhibition as a function of the relevance of vestibular information to the context. Specifically, in the sensorial stop-signal task participants needed to process vestibular information for positioning the Go stimulus with respect to their reference apparent zenith (RAZ). The RAZ is defined as the plan parallel to the direction of gravity and passing through the participant’s eyes. Internal representation of the RAZ is achieving by integrating direction of gravity and the body’s orientation relative to gravity. This is the result of central (cerebral) integration of bodily cues coming from various different sensory systems, notably the vestibular system^12^. Therefore, we assumed that participants processed vestibular information to provide response to the sensorial stop-signal task. The Go stimulus (a yellow dot) was located above or below the subject’s RAZ, with angle intervals of 1°, 2° and 3° (in absolute value) pseudo-randomized across all trials. By contrast, the classical stop-signal task did not require participants to process vestibular information to provide response; as in classical stop-signal task, they were instructed to respond to the shape of Go stimulus (here a ring or a circle).

### Data analysis

#### Standard measures of inhibition and decision-making

Classically, inhibition process is assessed by estimating the SSRT (inhibition latency) from the Go RT distribution^7,8,15^. One standard method, which refers to as the quantile method, has been shown to be reliable and robust against violations of assumptions underlying the horse race model^11,16^. Interestingly, the quantile method does not require an assumption of 50% inhibition as is the case in our data for the classical stop-signal task (P_inhib_ = [54–56%] at the group level). All RTs on correct Go-trials were arranged in ascending order, and the RT corresponding to the proportion of failed inhibition (1- P_inhib_) was selected. Then, the average SSD (calculated from all SSD values) was subtracted from the quantile RT, thus providing an estimate of SSRT. In this way, SSRT reflects the average time that the subject requires in order to successfully inhibit a motor response approximately 50% of the time. For assessment of decision-making process, standard behavioural metrics were RT and accuracy (percentage of Go-trials with correct responses, P_Go_).

#### Computational models of inhibition and decision-making dynamics

**Figure 2:**
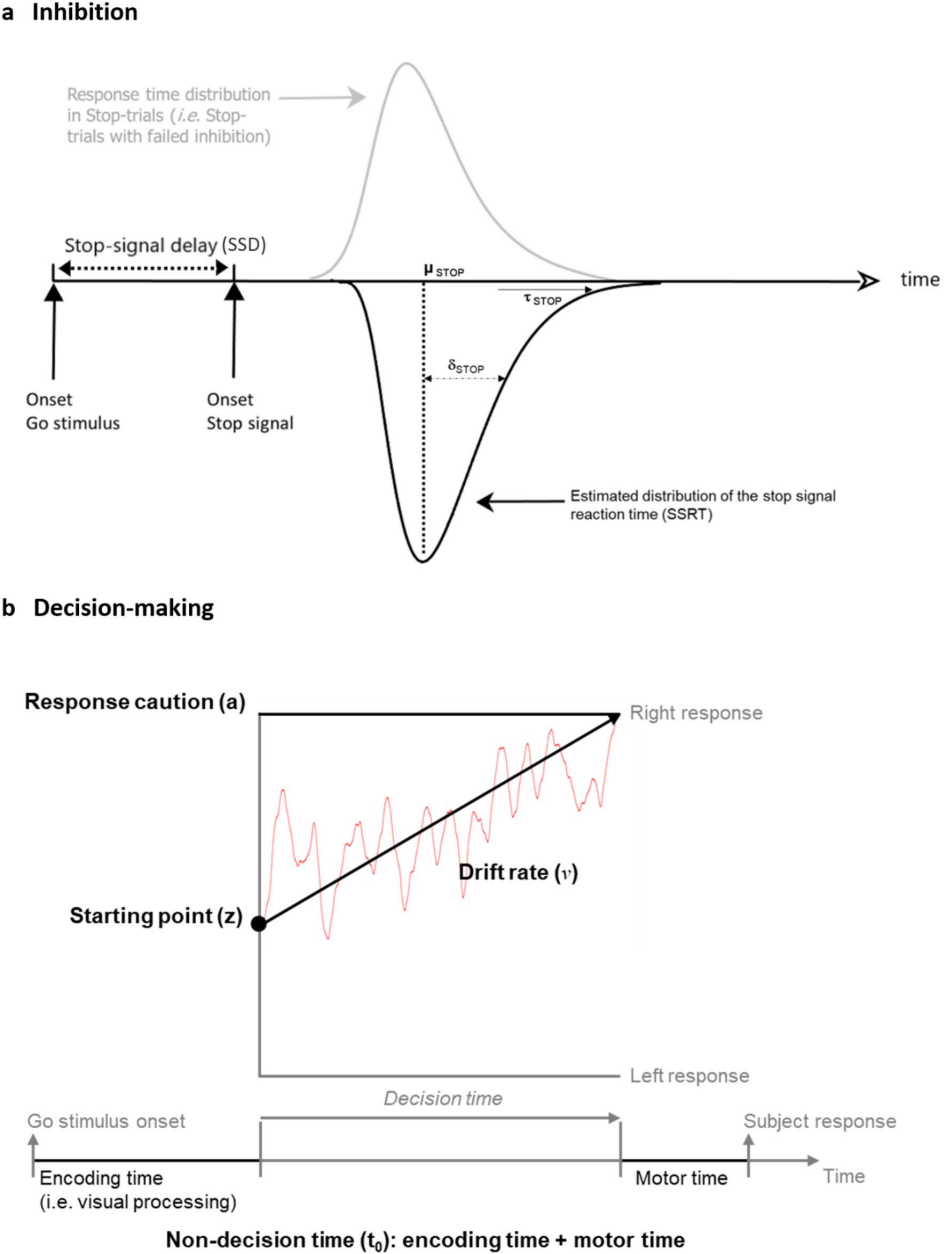
Computational models of inhibition and decision-making dynamics. (**a**) Graphical representation of the horse-race model of inhibition. In a Stop-trial, if the response time (RT) is slower than the sum of stop signal delay (SSD) and stop signal reaction time (i.e. inhibition latency, SSRT), the response is successfully inhibited. In contrast, if the RT is faster than SSD + SSRT, the response cannot be inhibited and results in a signal response RT (grey distribution). The model assumes that RTs and SSRTs are random variables. The distribution of RTs in Stop-trials (grey distribution) is seen as a RTs distribution that is censored by the SSRTs distribution. In the present study, three parameters of the SSRTs ex-Gaussian distribution were estimated: the mean (parameter μ_Stop_) and the standard deviation (parameter σ_Stop_) of the Gaussian component, and the mean (parameter τ_Stop_) of the exponential component. (**b**) Schematic representation of the diffusion decision model with evidence on the ordinate and time on the abscissa. Here four latent decision-making parameters were estimated: (i) the response caution (parameter *a*), which indicates the overall amount of evidence that needs to be accumulated before the choice is committed; (ii) the drift rate (parameter *ν*), which reflects the quality and strength of evidence from the stimulus; (iii) the non-decision time (parameter *t*_*0*_), which sums the duration of encoding and motor processes; and (iv) the starting point (parameter *z*), which refers to the response bias for one option over another (left *vs* right).

The aforementioned quantile method actually provides a summary measure of inhibition, i.e. a single SSRT value per participant, and thus may mask crucial features of experimental effects. To overcome this limitation, we also treated the SSRT as a random variable and its entire distribution was estimated using a Bayesian hierarchical computational approach (BHA). Of note, the BHA is well suited to handle experimental data with a small number of trials, as is the case in the present study because of the technical specifications of centrifuge-based design. The BHA used data from the entire group to estimate parameters at the individual level^17^. Three main parameters were estimated to characterize the SSRT distribution, given the BHA assumes that SSRTs follow an ex-Gaussian distribution: the *μ*_Stop_ and *σ*_Stop_ parameters give the mean and the standard deviation of the Gaussian component and reflect the leading edge and mode of the distribution, respectively; and the τ_Stop_ gives the mean of the exponential component and reflects the tail of the distribution (see Fig. 2a)^18^. Two additional parameters were estimated: the percent trigger-failures (TF) that quantifies failures to launch inhibition process in Stop-trials, and the percent Go-failures (GF) that accounts for errors of omission in the Go subtask. Estimating the TF and GF parameters has been shown to be critical to avoid over- or underestimation of SSRT distribution parameters, respectively^19–21^.

Parameters estimation in BHA relies on Markov Chain Monte Carlo (MCMC) sampling that yields posterior distribution for the model parameters. We refer the reader to the paper from Van Ravenzwaaij et al. (2018) for in‐depth coverage of the MCMC sampling method^22^. In the present work, BHA and MCMC sampling method were implemented using the R package *Dynamic Models of Choice* (DMC)^17^. We initialized MCMC sampling with 33 chains and 120 iterations for each chain. The effective sample size, which adjusts the actual sample size (33 chains $\times$ 120 samples) for redundancy due to autocorrelations, was around 250. We estimated the parameters described above for each condition and each task. Model convergence was assessed by applying both visual checking (MCMC chains should have the appearance of “flat fat hairy caterpillars”), the Gelman-Rubin statistic^23^, and several functions from the DMC package that quantify redundancy (autocorrelation) and the effective number of independent samples^17^.

Similarly, we used the BHA to estimate parameters of the DDM model characterizing decision-making in the Go subtask. Briefly, the DDM conceptualizes the decision-making process as an evidence accumulator governed by a diffusion process. It assumes that sensory evidence is accumulated over time until a decision threshold is reached, signalling commitment to that response option. Four main parameters relating to different cognitive components of decision-making were estimated: (i) the response caution (parameter *a*), a low value indicating that the subject does not accumulate many evidence before making a decision; (ii) the drift rate (parameter *ν*), which reflects the strength of evidence from the Go stimulus, thus informing the difficulty of the task; (iii) the non-decision time (parameter *t*_*0*_), which is the duration of encoding and motor processes; and (iv) the starting point (parameter *z*) that refers to a potential response bias for one option over another (see Fig. 2b)^10^. Of note, parameters of the DDM model have the potential to characterize individual differences in the decision making process with greater sensitivity than standard behavioural metrics (RT and P_Go_), and greater specificity for relating these differences to specific cognitive components of task performance^24^.

All parameters from computational models of inhibition and decision-making that were computed in the current study are summarized in Table 1.

**Table 1:** Parameters of computational models. Description of parameters from computational models of inhibition and decision-making that were computed in the current study.

Parameter	Working definition
**Inhibition**	
μ_Stop_	Mean of the main part (left part) of inhibition latency distribution
σ_Stop_	Standard deviation of the main part (left part) of inhibition latency distribution
τ_Stop_	Mean of the right part (tail) of inhibition latency distribution
**Decision-making**	
Response caution	How much evidence is needed to make a decision
Drift rate	How quickly evidence accumulates towards a decision threshold
Non-decision time	Duration of process outside the decision-making process (e.g., visual or motor process)
Starting point	How close the starting position is to one response and the other

### Statistical analysis

To test condition and task effects on inhibition and decision-making variables, we used both standard statistical tests and Bayesian equivalents to extend insight and guiding interpretation of significance (p values), according to how likely the alternative hypothesis is versus the null. Indeed, a disadvantage of null hypothesis significance testing is that non-significant p values (e.g., when reporting no condition effect on experimental measures) cannot be interpreted as support for the null hypothesis^25,26^. To circumvent this issue and confirm whether the potential non-significant findings reported represent support for the null hypothesis, we calculated the Bayes factor (BF): specifically, we computed the log scale of BF_10_ (noted log(BF_10_)) that can be easily interpreted such that a negative value indicates support for the null hypothesis, whereas a positive value indicates evidence in favour of the alternative hypothesis (see Supplementary Table 1 for an interpretation scale of log(BF_10_))^27^. Data were analysed in JASP (version 0.11.1, https://jasp-stats.org/). For standard post hoc tests we applied Holm correction for multiple comparisons. For the Bayesian analyses, we used the default JASP priors (paired samples t-tests: medium effect size on a Cauchy distribution of 0.707 ; repeated measures ANOVA: r scale fixed effects of 0.5, r scale random effects of 1, and r scale covariates of 0.354), and our model was compared to the null model for Bayesian repeated measures ANOVA^28^. Standard and Bayesian analyses were performed with either the experimental condition (condition effect) or the task (task effect) as a within-participants factor.

## Results

### Disrupted vestibular signal affects inhibition in increasing variability of longest inhibition latencies at the individual level

**Figure 3:**
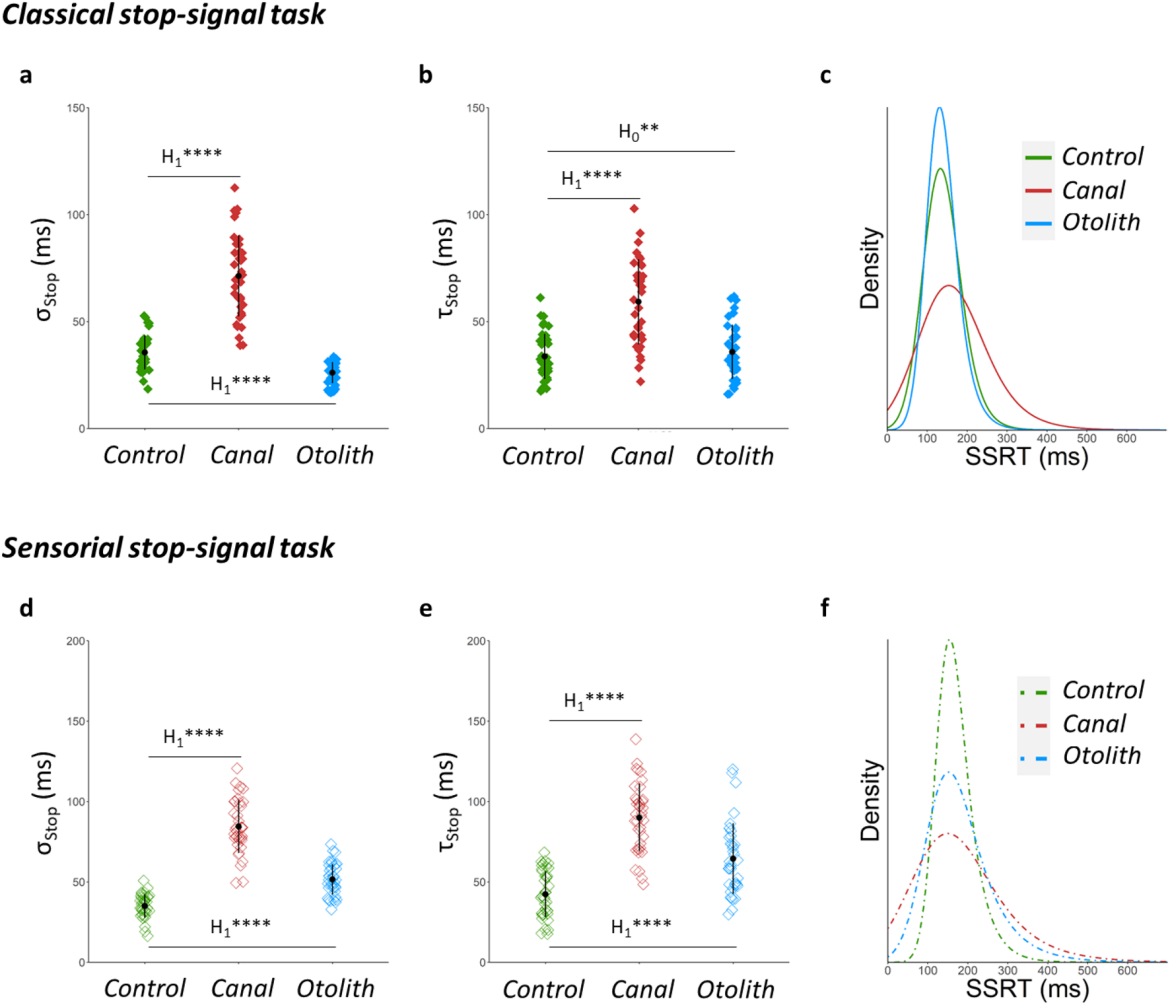
Effects of disrupted vestibular signal on inhibition performance according to computational measures. (**a–c**) In the classical stop-signal task, the stimulation of semicircular canals (condition Canal, in red) increases values of parameters σ_Stop_ and τ_Stop_ of SSRT distribution compared to condition Control (in green), as depicted by the expansion of the corresponding SSRT distribution tail. (**d**–**f**) In the sensorial stop-signal task, both the condition Canal and the condition Otolith (in blue), in which otoliths are selectively stimulated, are associated with higher values for parameters σ_Stop_ and τ_Stop_ of SSRT distribution, as illustrated by the slowing in the tail of their SSRT distributions. Working definition for parameters: σ_Stop_ is the standard deviation of the main part (left part) of SSRT distribution; τ_Stop_ is the mean of the right part (tail) of SSRT distribution. *Interpretation scale: H*_1_**** means extreme evidence for the alternative hypothesis; H_0_** means strong evidence for the null hypothesis.

When the semicircular canals were stimulated (condition Canal), the parameters σ_Stop_ and τ_Stop_ of SSRT distribution were shifted to higher values compared to control condition, both in the classical stop-signal task (parameter σ_Stop_: p ≤ 0.001, log(BF_10_) = 24.45, Fig. 3a; parameter τ_Stop_: p ≤ 0.001, log(BF_10_) = 18.90, Fig. 3b) and in the sensorial stop-signal task (parameter σ_Stop_: p ≤ 0.001, log(BF_10_) = 36.01, Fig. 3d; parameter τ_Stop_: p ≤ 0.001, log(BF_10_) = 11.21, Fig. 3e).

The selective stimulation of otoliths (condition Otolith) shifted parameters σ_Stop_ and τ_Stop_ of SSRT distribution to higher values in the sensorial stop-signal task (parameter σ_Stop_: p ≤ 0.001, log(BF_10_) = 21.41, Fig. 3d; parameter τ_Stop_: p ≤ 0.001, log(BF_10_) = 32.02, Fig. 3e), compared to control condition. In the classical stop-signal task, the parameter τ_Stop_ did not change (p = 0.43, log(BF_10_) = −1.28, Fig. 3b) and the parameter σ_Stop_ decreased (p ≤ 0.001, log(BF_10_) = 8.70, Fig. 3a) in condition Otolith compared to control condition.

To summarize, the conditions Canal and Otolith were characterized with increased value for the parameters σ_Stop_ and τ_Stop_ of SSRT distribution in the sensorial stop-signal task, illustrating that disruption of vestibular signal led to larger variability of inhibition latencies at the individual level. The aforementioned effect of increasing the value of parameters σ_Stop_ and τ_Stop_ was reflected in the slowing in the tail of the SSRT distribution (Fig. 3c and f). In other words, disruption of vestibular signal made the participants even slower to inhibit in the trials where they had the most difficulty inhibiting.

Furthermore, percentage of trigger failure (i.e. percentage of Stop signal missed by the subject, parameter TF^20^) was equal or lower when vestibular signal was disrupted compared to control condition, both in the sensorial stop-signal task (condition Canal: p = 0.08, log(BF_10_) = −0.66; condition Otolith: p ≤ 0.001, log(BF_10_) = 4.29) and in the classical stop-signal task (condition Canal: p ≤ 0.01, log(BF_10_) = 2; condition Otolith: p ≤ 0.05, log(BF_10_) = 0.47). Percentage of Go failure (i.e. percentage of Go stimuli missed by the subject, parameter GF^19^) was not affected by the experimental condition in the two tasks (classical stop-signal task: p = 0.27, log(BF_10_) = −1.38; sensorial stop-signal task: p = 0.09, log(BF_10_) = −0.41). To summarize, disrupted vestibular signal was not characterized with more frequent lapses of attention. This finding suggests that longer inhibition latencies reported in association with disrupted vestibular signal did not result from difficulty in perceiving the Stop signal.

Supplementary Table 2 (upper part) summarises statistics that inform the effects of disrupted vestibular signal on inhibition performance according to computational measures.

Standard measures of inhibition, including SSRT, SSD, and P_inhib_, did not reveal any effect of disrupted vestibular signal on inhibition performance (see Supplementary Table 3, upper part).

### Processing bodily information degrades response inhibition performance, all the more strongly when bodily signal is disrupted

**Figure 4:**
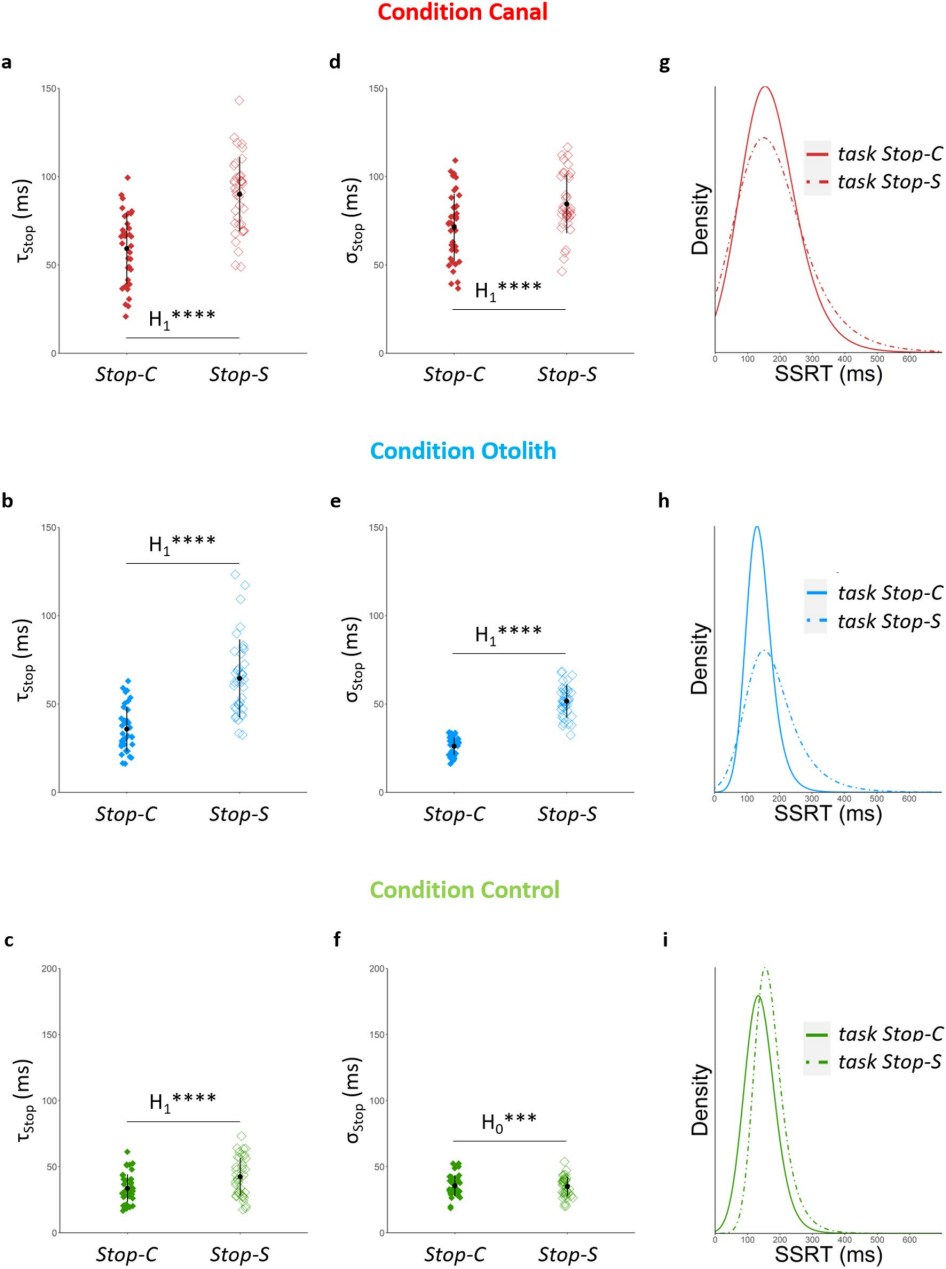
Effects of processing bodily information on inhibition performance according to computational measures. (**a**–**c**) In the sensorial stop-signal task (Stop-S), where individuals need to process bodily information, the value of parameter τ_Stop_ of SSRT distribution increases in all conditions, including control condition, compared to the classical stop-signal task (Stop-C). (**d**–**f**) The sensorial stop-signal task is also associated with increased values for the parameter σ_Stop_ of SSRT distribution when vestibular signal is disrupted (conditions Canal and Otolith), as opposed to the condition Control (**g**–**i**) In conditions Canal and Otolith, combination of increased values for parameters τ_Stop_ and σ_Stop_ is depicted by the expansion of the corresponding SSRT distribution tail, which is less marked in the condition Control where only the parameter τ_Stop_ significantly increases. Working definition for parameters: σ_Stop_ is the standard deviation of the main part (left part) of SSRT distribution; τ_Stop_ is the mean of the right part (tail) of SSRT distribution. *Interpretation scale: H*_1_**** means extreme evidence for the alternative hypothesis; H_0_*** means very strong evidence for the null hypothesis.

When processing information from the body (sensorial stop-signal task), SSRT distribution was consistently characterized by the increase of parameter τ_Stop_ in all experimental conditions including control condition, namely regardless of whether the vestibular signal was disrupted (condition Canal: p ≤ 0.001, log(BF_10_) = 21.83, Fig. 4a; condition Otolith: p ≤ 0.001, log(BF_10_) = 16.90, Fig. 4b; condition Control: p ≤ 0.001, log(BF_10_) = 4.53, Fig. 4c). In addition, when vestibular signal was disrupted (conditions Canal and Otolith), the parameter σ_Stop_ was also shifted to higher values for the sensorial stop-signal task relative to the classical stop-signal task (condition Canal: p ≤ 0.01, log(BF_10_) = 2.97, Fig. 4d; condition Otolith: p ≤ 0.001, log(BF_10_) = 36.01, Fig. 4e), as opposed to the control condition where the parameter σ_Stop_ did not differ between the two tasks (p = 0.76, log(BF_10_) = −1.70, Fig. 4f). To summarize, processing of bodily information, as required in the sensorial stop-signal task, was characterized by increased value of the parameter τ_Stop_ of SSRT distribution. Furthermore, when the vestibular signal was disrupted (conditions Canal and Otolith), the inhibition pattern of the sensorial stop-signal task in addition was associated with increased value of the parameter σ_Stop_ of SSRT distribution. Such a combination of increased values for parameters τ_Stop_ and σ_Stop_ is reflected in the slowing in the tail of the SSRT distribution (Fig. 4g,h). This finding illustrates that processing bodily information leads to a larger variability of longest inhibition latencies at the individual level, which is all the more important when vestibular signal is disrupted. In other words, disruption of vestibular signal makes individuals even slower to inhibit in the trials where they have to process bodily information.

The sensorial stop-signal task, compared to the classical stop-signal task, shifted the parameter μ_Stop_ of SSRT distribution to lower values in conditions Canal and Otolith (condition Canal: p ≤ 0.001, log(BF_10_) = 11.41; condition Otolith: p ≤ 0.001, log(BF_10_) = 4.81), and to higher values in the condition Control (p ≤ 0.001, log(BF_10_) = 17.61). Standard measure of inhibition accuracy (P_inhib_) was consistently lower in the sensorial stop-signal task compared to the classical stop-signal task (condition Control: p ≤ 0.01, log(BF_10_) = 2.67; condition Canal: p ≤ 0.001, log(BF_10_) = 4.65; condition Otolith: p ≤ 0.05, log(BF_10_) = 1.01). To summarize, processing bodily information (as required in the sensorial stop-signal task) while the vestibular signal was disrupted (conditions Canal and Otolith) resulted in shorter inhibition latencies in combination with lower inhibition accuracy. In other words, a shorter inhibition latency did not necessarily imply a better response inhibition performance. According to the horse race model, such an event may occur if a very fast Go process precedes the Stop process because shortening inhibition latency could be insufficient to allow the Stop process to finish before the very fast Go process. To test this hypothesis, we have investigated the decision-making process related to the Go stimulus.

Supplementary Table 2 (lower part) and Supplementary Table 3 (lower part) summarise statistics that inform the task effect (classical stop-signal task *vs* sensorial stop-signal task) on inhibition performance according to computational measures and standard measures, respectively.

### Processing bodily information speeds up the decision-making with a less conservative decision threshold and a reduced non-decisional time

Participants showed faster responses in the sensorial stop-signal task than in the classical stop-signal task, in all experimental conditions (condition Control: p ≤ 0.01, log(BF_10_) = 2.88; condition Canal: p ≤ 0.01, log(BF_10_) = 2.91; condition Otolith: p ≤ 0.05, log(BF_10_) = 1.1). Specifically, participants responded faster in the sensorial stop-signal task when the Go stimulus was presented away from their RAZ (i.e. angle intervals of −3°, −2°, 2° and 3°; see Supplementary Tables 5 and 6). Furthermore, participants showed a lower accuracy on Go trials in the sensorial stop-signal task compared to the classical stop-signal task (condition Control: p ≤ 0.001, log(BF_10_) = 9.79; condition Canal: p ≤ 0.001, log(BF_10_) = 7.28; condition Otolith: p ≤ 0.001, log(BF_10_) = 5.61). The within-individual variability in RTs (IIV RT) was consistently larger in the sensorial stop-signal task than in the classical stop-signal task (condition Control: p ≤ 0.001, log(BF_10_) = 11.51; condition Canal: p ≤ 0.01, log(BF_10_) = 2.03; condition Otolith: p ≤ 0.001, log(BF_10_) = 15.98). In summary, the sensorial stop-signal task was characterized with a shorter decision-making process, a lower accuracy and an increase in the variability of RTs. These results illustrate that the sensorial stop-signal task was more difficult but it was associated with a shortening of the decision-making process (specifically for the Go subtask, which required participants to process bodily information for positioning a stimulus with respect to their body position). In other words, participants responded quickly despite the high level of difficulty of the task. Supplementary Table 4 (lower part) summarises statistics that inform the task effect (classical stop-signal task vs sensorial stop-signal task) on decision-making variables according to standard measures.

**Figure 5:**
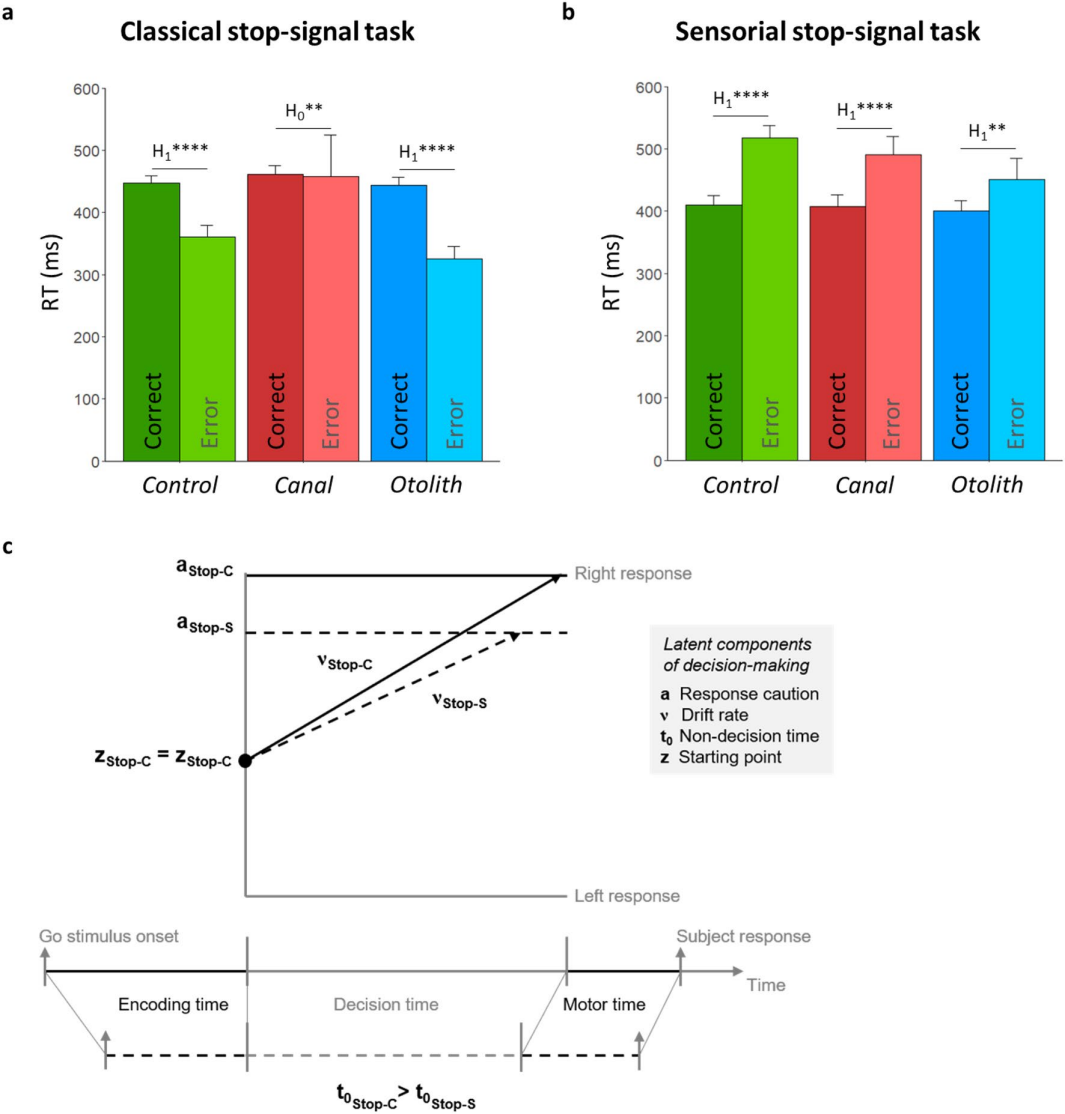
Effects of processing bodily information on decision-making performance. (**a**) On the classical stop-signal task (Stop-C), errors (lighter colors) are faster than correct responses (darker colors), except on the condition Canal where no difference was found. (**b**) By contrast, on the sensorial stop-signal task (Stop-S), errors are consistently slower than correct responses. (**c**) Schematic representation of the task effect on the parameters of diffusion decision model (solid black lines refer to the classical stop-signal task, and dotted black lines refer to the sensorial stop-signal task). Working definition for parameters: Response caution: how much evidence is needed to make a decision; Drift rate: how quickly evidence accumulates towards a decision threshold; Non-decision time: duration of process outside the decision-making process (e.g., visual or motor process); Starting point: How close the starting position is to one response and the other. *Interpretation scale: H*_1_**** means extreme evidence for the alternative hypothesis; H_0_** means strong evidence for the null hypothesis.

The nature of trial response (correct *vs* error) interacted with the effect of task on RTs: on the classical stop-signal task, errors were faster than correct responses (condition Control: p ≤ 0.001, log(BF_10_) = 4.04; condition Otolith: p ≤ 0.001, log(BF_10_) = 3.74), except on the condition *Canal* where no difference was found (condition Canal: p = 0.84, log(BF_10_) = −1.48) (Fig. 5a); by contrast, on the sensorial stop-signal task, errors were consistently slower than correct responses (condition Control: p ≤ 0.001, log(BF_10_) = 13.51; condition Canal: p ≤ 0.001, log(BF_10_) = 3.79; condition Otolith: p ≤ 0.05, log(BF_10_) = 1.01; Fig. 5b). These results mean that participants favoured accuracy on speed as response strategy in the sensorial stop-signal task, whereas they sacrificed accuracy for speed in the classical stop-signal task. Supplementary Table 7 summarises statistics that inform the effect of nature of trial response (correct *vs* error) on RTs.

For DDM-based latent components of decision-making, parameters *a* (response caution), *t*_*0*_ (non-decision time), and ν (drift rate) consistently shifted to lower values for the sensorial stop-signal task relative to the classical stop-signal task (parameter *a*: p ≤ 0.001 and log(BF_10_) ≥ 22.91 in all conditions; parameter *t*_*0*_: p ≤ 0.001 and log(BF_10_) ≥ 8.96 in all conditions; parameter ν: p ≤ 0.001 and log(BF_10_) ≥ 23.97 in all conditions; Fig. 5c). The parameter *z* (starting point) did not differ between the two tasks (p ≥ 0.14 and log(BF_10_) ≤ −0.7 in all conditions; Fig. 5c). To summarize, in the sensorial stop-signal task, participants made a decision more quickly even when strength of sensory evidence was reduced. Such behaviour, in combination with shorter non-decision time (visual processing and motor time), contributes to explain decreased reaction times in the sensorial stop-signal task. Supplementary Table 8 (lower part) summarises statistics that inform the task effect (classical stop-signal task *vs* sensorial stop-signal task) on the four DDM-based latent parameters of decision-making.

Descriptive statistics (mean and standard deviation) of all inhibition and decision-making variables are summarized in Tables 2 and 3, respectively, for each task and each condition separately.

**Table 2:** Inhibition performance. Descriptive statistics (mean ± standard deviation) of inhibition measures, including standard metrics (upper part) and parameters estimated using a computational-based approach (lower part), as a function of the task and the condition.

Variable (unit)	Classical stop-signal task			Sensorial stop-signal task		
	Condition			Condition		
	Control	Otolith	Canal	Control	Otolith	Canal
P_Inhib_ (%)	54 ± 6	55 ± 7	56 ± 7	50 ± 7	51 ± 11	50 ± 10
SSRT (ms)	157 ± 41	159 ± 36	165 ± 49	171 ± 44	169 ± 50	179 ± 59
SSD (ms)	268 ± 66	258 ± 71	262 ± 64	218 ± 74	206 ± 77	200 ± 65
µ_Stop_ (ms)	109 ± 11	112 ± 8	106 ± 9	126 ± 15	105 ± 9	89 ± 19
σ_Stop_ (ms)	36 ± 8	26 ± 5	71 ± 19	35 ± 7	52 ± 9	84 ± 17
τ_Stop_ (ms)	34 ± 11	36 ± 13	59 ± 20	42 ± 14	64 ± 22	90 ± 21
TF (%)	5.13 ± 6.08	2.70 ± 2.72	1.91 ± 2.45	3.38 ± 3.70	1.05 ± 0.53	4.37 ± 2.29
GF (%)	0.28 ± 0.64	0.26 ± 0.86	0.52 ± 1.11	1.17 ± 1.21	0.63 ± 1.47	1.16 ± 1.39

P_Inhib_: inhibition accuracy (percentage of Stop-trials with successful inhibition); SSRT: stop signal reaction time; ms: milliseconds; SSD: stop signal delay; µ_Stop_: mean of the Gaussian component of the SSRTs distribution; σ_Stop_: standard deviation of the Gaussian component of the SSRTs distribution; τ_Stop_: mean of the exponential component of the SSRTs distribution; GF: Go failure (percentage of Go stimuli missed by the subject); TF: trigger failure (percentage of stop-signal missed by the subject).

**Table 3:** Decision-making performance. Descriptive statistics (mean ± standard deviation) of measures that inform the decision-making process, including standard behavioural metrics (upper part) and four main parameters of the diffusion decision model (lower part), as a function of the task and the condition.

Variable (unit)	Classical stop-signal task			Sensorial stop-signal task		
	Condition			Condition		
	Control	Otolith	Canal	Control	Otolith	Canal
P_Go_ (%)	98 ± 2	98 ± 4	97 ± 3	91 ± 6	90 ± 10	91 ± 7
RT_Tot_ (ms)	446 ± 77	442 ± 85	461 ± 88	417 ± 93	407 ± 107	416 ± 112
RT_Correct_ (ms)	447 ± 77	443 ± 85	461 ± 86	410 ± 94	401 ± 103	408 ± 113
RT_Error_ (ms)	361 ± 88	326 ± 70	457 ± 294	518 ± 121	450 ± 191	490 ± 174
IIV RT (ms)	105 ± 22	96 ± 24	115 ± 50	138 ± 31	138 ± 34	153 ± 60
*a* (a.u.)	1.78 ± 0.18	1.73 ± 0.16	1.74 ± 0.13	1.35 ± 0.21	1.33 ± 0.24	1.26 ± 0.15
*ν* (a.u.)	4.00 ± 0.44	4.20 ± 0.46	3.91 ± 0.36	1.49 ± 1.44	1.26 ± 1.40	1.43 ± 1.23
t_0_ (ms)	224 ± 68	238 ± 77	239 ± 62	151 ± 78	151 ± 95	143 ± 85
*z* (a.u.)	0.51 ± 0.05	0.51 ± 0.06	0.51 ± 0.06	0.52 ± 0.07	0.50 ± 0.06	0.51 ± 0.07

P_Go_: decision-making accuracy (percentage of Go-trials with correct responses); RT_Tot_: mean reaction time for all Go-trials; ms: milliseconds; RT_Correct_: mean reaction time for Go-trials with correct responses; RT_Error_: mean reaction time for Go-trials with errors; IIV RT: intra-individual variability in reaction times; *a*: response caution ; a.u.: arbitrary unit; ν: drift rate; *t*_*0*_: non-decision time; z: starting point SSRT: stop signal reaction time; ms: milliseconds; SSD: stop signal delay; µ_Stop_: mean of the Gaussian component of the SSRTs distribution; σ_Stop_: standard deviation of the Gaussian component of the SSRTs distribution; τ_Stop_: mean of the exponential component of the SSRTs distribution; GF: Go failure (percentage of Go stimuli missed by the subject); TF: trigger failure (percentage of stop-signal missed by the subject).

## Discussion

### Disrupted vestibular signal impairs inhibition

The first major observation of the present study is that the disruption of vestibular signal degrades the response inhibition performance. Using a centrifuge-based design that disrupted vestibular system through short-term gravitational alteration, we found an increase in the variability of inhibition latencies at the individual level. When stimulating semicircular canals, the parameters σ_Stop_ and τ_Stop_ of SSRT distribution increased, illustrating that longest inhibition latencies were characterized by a larger variability. Furthermore, the selective stimulation of otoliths impaired inhibition as a function of the relevance of otolith information to the context. We used two different tasks to manipulate the relevance of vestibular information, and disrupted otolith signal only affected inhibition when processing otolith information was required to solve the Go subtask. In this respect, parameters σ_Stop_ and τ_Stop_ of SSRT distribution increased, thus illustrating once again a larger variability of the longest inhibition latencies. It has been suggested that the tail of SSRT distribution indicates lapses of attention rather than inhibitory capacity^29,30^. Here, we found that slowing in the tail of SSRT distribution can be associated with less failure to launch inhibition process (parameter TF), thus suggesting that impaired inhibition caused by disrupted vestibular signal is not the result of attentional deficit. To summarize, our results demonstrate that disrupted vestibular signal degrades inhibition depending on the relevance of vestibular information to the context. The present study is novel in that, to date, it is the first work to examine the influence of *exteroceptive* signals on inhibition. Our findings are in line with the recent work of Rae et al*.* focusing on interoception that has shown that cardiac signal influences response inhibition performance^1^. Our results also extend previous research showing that exteroceptive signals influence spatial cognitive ability, in particular when they are disrupted such as in sensory illusions (e.g. the somatogravic illusion^31,32^, the Aubert effect^33–35^). Of note, in the present study, standard analyses of inhibition (which yield a summary measure of the inhibition latency at the individual level, specifically a mean SSRT per participant) did not reflect the significant effect of disrupted vestibular signal on inhibition performance. It suggests that important features of the data may be missed in focusing only on the mean. Interestingly, model-bases analyses of inhibition, which reveal the shape of the entire SSRT distribution, have the potential to provide greater sensitivity for observing individual differences in inhibition response performance.

Our insights have substantial implications for environments where individuals are exposed to high body constraints and cognitively demanding situations. Our findings suggest that disruption of vestibular signal through short-term gravitational alteration, which may characterize some phases of aerobatic or military flight, might impair crew’s response inhibition capacity. Short-term gravitational alteration resulting in disrupted vestibular signal may also characterize space flights, for example during launch and entry phases of spacecraft^36,37^. Interestingly, it is proposed that artificial gravity could be an effective countermeasure to minimizing side effects of spaceflight environment due to microgravity^37^. We here provide evidence that gravitational alteration during intermittent artificial gravity might actually impair crew’s cognition (at least transiently), thus suggesting that concurrent countermeasure might be necessary to optimize artificial gravity-based countermeasure during long-duration space flights.

### Processing information from the body impairs inhibition, particularly when bodily signal is disrupted

We further found that inhibition was impaired when processing bodily information was required to solve the Go subtask (sensorial stop-signal task), with a decreased inhibition accuracy and a larger variability of the longest inhibition latencies (increased parameter τ_Stop_ of SSRT distribution). Importantly, this effect was observed in all conditions including the control condition, namely regardless of whether the vestibular signal was disrupted. In addition, when the vestibular signal was disrupted (conditions Canal and Otolith), the inhibition pattern of the sensorial stop-signal task was characterized with an even greater increase in variability of longest inhibition latencies (due to increase in the parameter σ_Stop_ of SSRT distribution). Taken together, our findings demonstrate that processing information from the body degrades inhibition, particularly when bodily signal is disrupted. In addition, results show that the nature of the Go subtask influences the inhibition performance and therefore support the idea that the Go and Stop processes are not completely independent^9^. The novel, sensorial stop-signal task we have developed extends the assessment of inhibition to situations where response that needs to be suppressed stems from processing of exteroceptive information. This task is particularly attractive because it might provide a more ecologically valid test of inhibition, since most of actions that are inhibited in daily life involve bodily information processing (e.g. inhibiting a movement of foot while walking). And, more importantly, the sensorial stop-signal task allows a sophisticated understanding of how inhibition is influenced by exteroceptive signal. In particular, it provides a way to test whether exteroceptive signal affects inhibition as a function of the relevance of exteroceptive information to the context. The findings of the present study are consistent with the recent work of Kunzendorf et al*.* focusing on interoception that has shown that cardiac signal differentially modulates the processing of information according to its relevance to the context^38^.

Furthermore, we found that mean inhibition latency (parameter μ_Stop_ of SSRT distribution) decreased when the vestibular signal was disrupted (conditions Canal and Otolith) while processing bodily information (sensorial stop-signal task). Interestingly, such an observation conflicts with the lower inhibition accuracy we reported above. Indeed, according to the horse race model, a decreased mean inhibition latency that reflects a faster Stop process should lead to a higher level of inhibition success, given the Stop process is much more likely to finish before the Go process^7,8^. Alternatively, if a very fast Go process precedes the Stop process, shortening the inhibition latency could be insufficient to maintain the level of inhibition success. Consequently, a decreased mean inhibition latency can be associated with a lower (or the same) inhibition performance due to the high speed of Go stimulus processing. To test this latter hypothesis and disentangling apparently contradictory results regarding the inhibition performance, the decision-making process related to the Go stimulus needs to be investigated.

### Processing bodily information speeds up the decision-making process and ultimately leads to poor cognitive performance

Overall, almost all participants reported that the sensorial Go subtask, which required them to process bodily information for positioning the stimulus with respect to their RAZ, was more difficult than the classical one. Standard behavioural measures supported participants’ subjective reports in showing a lower decision-making accuracy (P_Go_) and a larger within-individual variability in RTs (IIV RT), in the sensorial stop-signal task compared with the classical stop-signal task. In addition, the latent decision-making component of drift rate (parameter ν), which reflects the quality and strength of evidence from the Go stimulus, was lower in the sensorial stop-signal task thus supporting the higher level of difficulty of this task. Surprisingly, even though processing bodily information was more difficult, it was not corroborated by a lengthening of the decision-making process. Rather, RTs were shorter or equal in the sensorial stop-signal task compared with the classical stop-signal task, depending on the angle interval of the Go stimulus from the RAZ. First and foremost, the combination of high error rates and short RTs might suggest that participants made more errors in the sensorial stop-signal task because they responded quickly. The speed-accuracy trade-off can be tested by investigating RTs as a function of the response: error responses are typically faster than correct responses when speed is stressed and are usually slower than correct responses when accuracy is stressed^10^. We found that error responses were consistently slower than correct responses in the sensorial stop-signal task, thus rejecting the hypothesis that participants committed more errors because they responded quickly. To summarize, processing information from the body speeds up the decision-making process although participants perceived the task as more difficult and favoured an accuracy-based response strategy. To clarify these counterintuitive findings, the decision-making process may be probed more deeply using the DDM-based approach. Of note, the DDM-based latent components that are computationally estimated from behavioural data are immune to speed-accuracy trade-offs. We found that the shortening of decision-making process affected both its decisional and non-decisional components. Specifically, the response caution (parameter a), which indicates the overall amount of sensory evidence that needs to be accumulated before decision, was lower in the sensorial stop-signal task compared to the classical stop-signal task. In other words, participants accumulated less evidence when processing bodily information before deciding on one or the other response alternative. Thus, participants likely failed to maintain the level of success in the Go subtask because their choice was committed long before enough discriminative bodily evidence was accumulated to respond accurately. Previous research in clinical population, including vestibular patients, has suggested that disrupted vestibular signal could slow decision-making process by reducing strength of sensory evidence (*i.e.*, a low value for the drift rate parameter)^39^. In healthy population, previously published works have shown that interoceptive signal, for example cardiac systole, facilitates spontaneous or self-initiated motor actions^38,40,41^. More recently, Rae et al. have reported mixed findings regarding the impact of cardiac signal on intentional inhibition: although decisions to make or withhold actions seemed not to be influenced by cardiac phase, lower insight into bodily signals was linked to urges to move the body. The authors have suggested that reactive behaviour might result from noisy evidence that increases drift rate, and tips accumulators for execution of action towards motor threshold^42^. Interestingly, our results shed light on an alternative hypothesis in providing experimental evidence that processing bodily information might foster reactive behaviour through a less conservative response criterion for decision-making.

To summarize, our findings provide direct evidence that processing information from the body degrades the decision-making and the response inhibition. Specifically, the processing of bodily information is associated with a shortening of the decision-making process, which results from a less conservative decision threshold and a reduced non-decisional (encoding and motor) time. Consequently, individuals fail to maintain a given level of inhibition success because a faster decision-making process requires a greater strength of inhibitory control for successfully inhibiting the response.

To improve and extend the understanding of how bodily information influences cognition, further experiments should investigate interoceptive and exteroceptive signals in a unified design because their combined effects on cognition are difficult to anticipate. For example, returning to the implications of present findings for the future of human space flights, the combined investigation of exteroceptive and interoceptive signals could provide a mean to disentangle the effect of gravitational alteration from potential confounding factors such as stress. Indeed, it is well known that stress response induces physiological changes and may also affect cognition^43^. In the present study, data from 16 participants were discarded before formal analyses because they did not meet recommended criteria for reliable estimate of inhibition latency^11^. Interestingly, most of participants that were excluded showed excessive inhibition performance that was not associated with excessive Stop signal failure (i.e. Stop signal missed by the participant). In other words, excessive inhibition performance appears not to result from failure of attention to the Stop signal, thus suggesting that the stop-signal task was performed appropriately. One potential explanation is that centrifuge rotation may provide some participants with very uncommon experience, potentially causing stress, which could ultimately result in unusual cognitive performance. Even though our experimental protocol originally included a training session, it may be insufficient to familiarize all the participants with rotating centrifuge. Furthermore, a “holistic”, multisignal approach could provide ecologically valid conditions to properly investigate mechanisms by which the brain integrates information originating within and outside the body, including physiological signals of stress response. Some additional physiological signals might also be particularly informative to provide a fined-grained analysis of how cognitive functions are affected by (disrupted) bodily information. Thus, electroencephalographic and electromyographic signals can inform about central and peripheral responses (e.g., error related negativity, partial error, etc.) to environment-body-brain interactions, beyond standard behavioural settings. Yet, some technical challenges should be addressed beforehand, particularly in centrifuge-based designs because of technical specifications resulting from rotation and the limited space on platform, to answer this promising question for neuroscience.

### Ethics statement

The study has been approved by the Ile de France XI independent ethics committee (2019-A01212-55) and was conducted in accordance with ethical standards of the 1964 Helsinki declaration and its later amendments.

### Informed consent

Written informed consent was obtained from all individual participants included in the study.

## Supplementary Information

Supplementary Information.

## Data Availability

All requests for raw and analyzed data should be addressed to dcssa-paris@sante.defense.gouv.fr, because they will be reviewed by our legal department (French Military Health Service) to verify whether the request is subject to any confidentiality constraints. Requests regarding materials, including programming code, should be addressed to the corresponding author (CV).
