# Supplementary material for: Environment and body-brain interplay affect inhibition and decision-making
Source: Sci Rep. 2022 Mar 11;12:4303. doi: 10.1038/s41598-022-08280-3 (PMC8917140; doi:10.1038/s41598-022-08280-3)
Supplement: Supplementary file 1 — Supplementary Information. [file 41598_2022_8280_MOESM1_ESM.docx]

**Supplementary Information**

**Title**

Environment and body-brain interplay affect inhibition and decision-making

**Authors and affiliations**

Pierre BOUNY^(a)^, Marion TROUSSELARD^(a,b,c)^, Sandrine JACOB^(a)^, François VIALATTE^(d)^, Charles VERDONK^(a,d,*)^

1. French Armed Forces Biomedical Research Institute

Department of Neurosciences and cognitive sciences

Unit of Neurophysiology of stress

91220 Brétigny-sur-Orge, France

1. French Military Health Service Academy

75005 Paris, France

1. University of Lorraine

APEMAC-EPSAM EA 4360

57006 Metz, France

1. ESPCI Paris – PSL University

75005 Paris, France

**^*^Corresponding author:**

Charles VERDONK

Mail: verdonk.charles@gmail.com

**
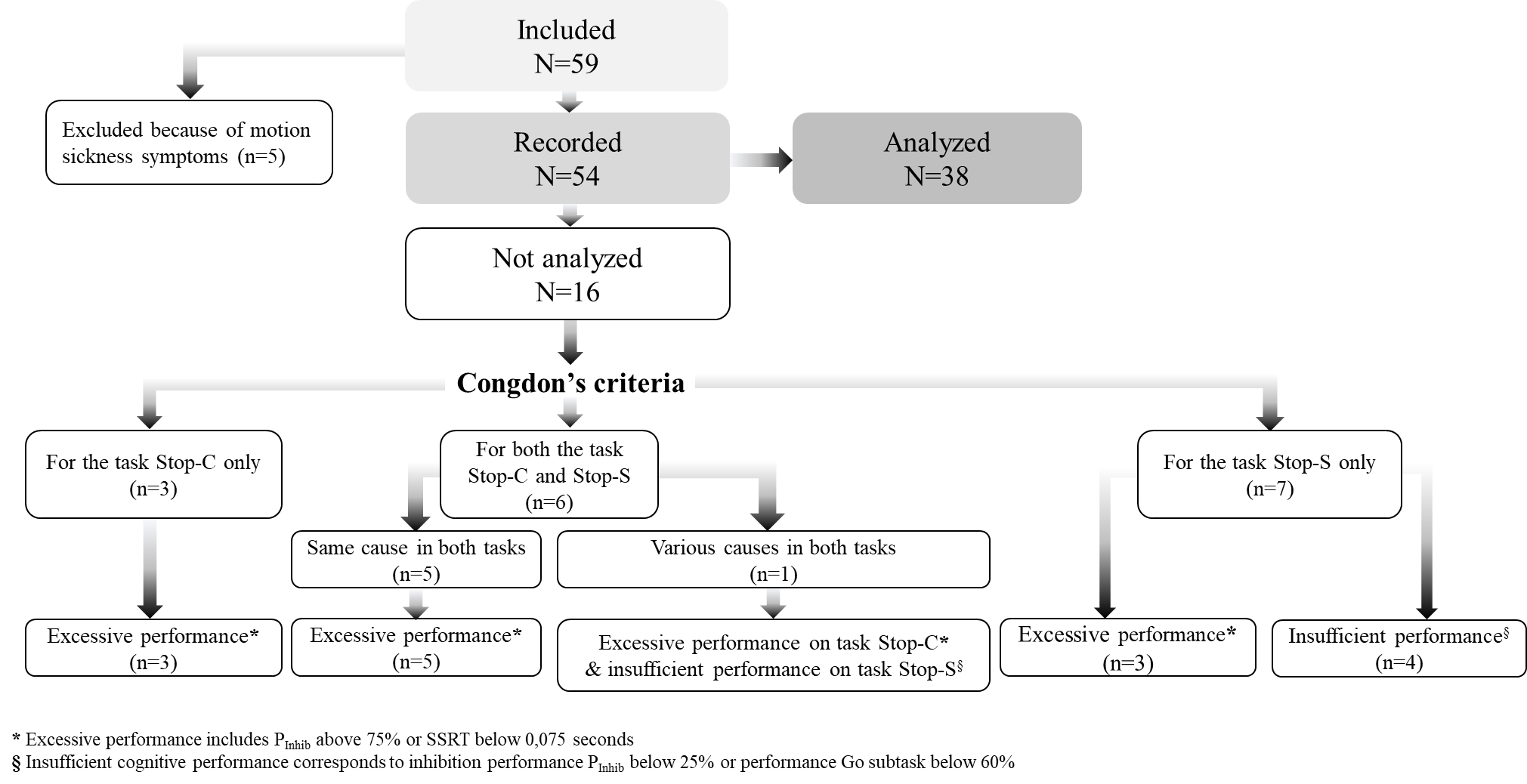
**

**Supplementary Figure 1 –** Flow chart of participants. Task Stop-C: classical stop-signal task; task Stop-S: sensorial stop-signal task.

**
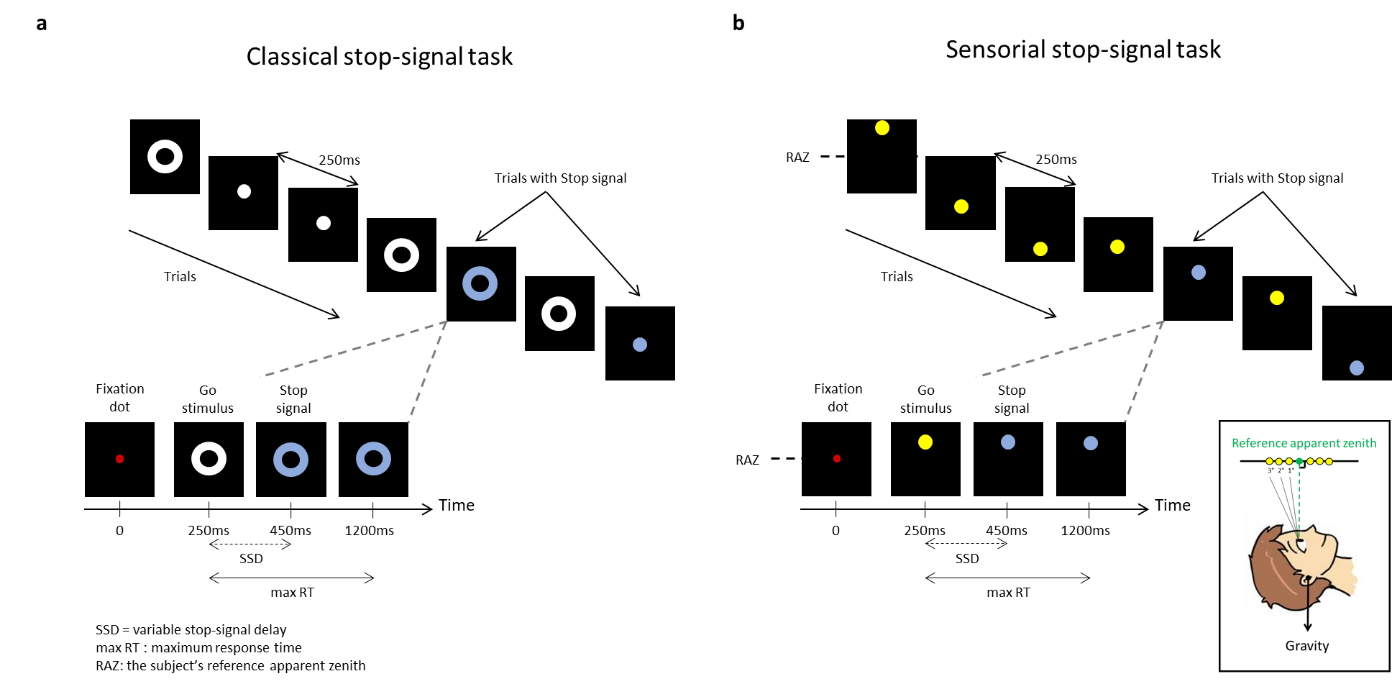
**

**Supplementary Figure 2 – The Stop-signal paradigm**. Display sequences for **(a)** the classical and **(b)** the sensorial stop-signal tasks. In both tasks, participants respond to a Go stimulus, and in a minority of trials (30%) the Go stimulus is followed by a Stop signal that instructs participants to withhold their response. The two tasks differed by the nature of the Go stimulus, which allows us to test whether exteroceptive signal influences inhibition as a function of the relevance of exteroceptive information to the context*.* In the sensorial stop-signal task **(b)**, participants need to process exteroceptive information for positioning the Go stimulus with respect to their reference apparent zenith (RAZ). The RAZ is the plane through eyes that the subject perceived as being parallel to the direction of gravity. The Go stimulus was located above or below the subject’s RAZ, with angle intervals of 1°, 2° and 3° (in absolute value) pseudo-randomized across all trials. By contrast, the classical stop-signal task **(a)** does not require participants to process exteroceptive information to provide response, and the Go stimulus was a geometric shape as in the classical stop-signal paradigm.

**Supplementary Table 1 -** **Bayes factor interpretation**. A descriptive and approximate classification scheme for the interpretation of the log scale of Bayes factor BF_10_ (adapted from Jeffreys, 1961).

**
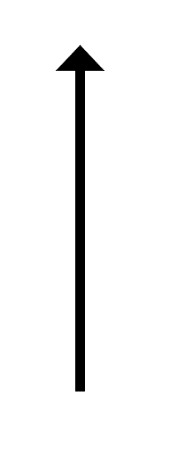

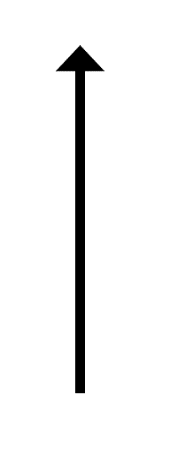
**

Growing evidence in favoutr of H_0_

Growing evidence in favoutr of H_1_

| **Log (BF_10_)** | **Interpretation** | **Symbol** |
| --- | --- | --- |
|  |  |  |
| > 2 | extreme evidence for H_1_ | H_1_**^****^** |
| [1.48 ; 2] | very strong evidence for H_1_ | H_1_**^***^** |
| [1 ; 1.48] | strong evidence for H_1_ | H_1_^**^ |
| [0.48 ; 1] | moderate evidence for H_1_ | H_1_^*^ |
| [0 ; 0.48] | anecdotal evidence for H_1_ | ns |
| 0 | no evidence | ns |
| [-0.48 ; 0] | anecdotal evidence for H_0_ | ns |
| [-1 ; -0.48] | moderate evidence for H_0_ | H_0_**^*^** |
| [-1.48 ; -1] | strong evidence for H_0_ | H_0_**^**^** |
| [-2 ; -1.48] | very strong evidence for H_0_ | H_0_**^***^** |
| < -2 | extreme evidence for H_0_ | H_0_**^****^** |

log(BF_10_): log scale of Bayes factor BF_10_; H_1_: alternative hypothesis; ns: non-significant; H_0_: null hypothesis

**Supplementary Table 2 - Condition and task effects on inhibition performance according to computational measures.** Standard statistics and Bayesian equivalents inform the effects of condition (upper part) and task (lower part) on computational parameters of inhibition.

|  |  |  | Computational measures of inhibition | | | | | | | | | | | | | | |
| --- | --- | --- | --- | --- | --- | --- | --- | --- | --- | --- | --- | --- | --- | --- | --- | --- | --- |
|  |  |  | **μ_Stop_** | | | **σ_Stop_** | | | **τ_Stop_** | | | **TF** | | | **GF** | | |
|  |  |  | *Stat* | *p* | *BF* | *Stat* | *p* | *BF* | *Stat* | *p* | *BF* | *Stat* | *p* | *BF* | *Stat* | *p* | *BF* |
| Effect of condition | **Task Stop-C** | *ANOVA* | 8.21 | <0.001 | 3.89 | 149.9 | <0.001 | 70.28 | 58.35 | <0.001 | 31.23 | 6.37 | <0.01 | 3.37 | 1.32 | 0.27 | -1.38 |
|  |  | *Post-hoc tests* |  |  |  |  |  |  |  |  |  |  |  |  |  |  |  |
|  |  | Control *vs* Otolith | -2.11 | 0.08 | -0.16 | 3.44 | <0.001 | 8.70 | -0.80 | 0.43 | -1.28 | 2.58 | <0.05 | 0.47 | 0.09 | 0.93 | -1.74 |
|  |  | Control *vs* Canal | 1.94 | 0.08 | 0.09 | -12.9 | <0.001 | 24.45 | -9.73 | <0.001 | 18.90 | 3.42 | <0.01 | 2.00 | -1.36 | 0.46 | -1.04 |
|  |  | Otolith *vs* Canal | 4.05 | <0.001 | 5.79 | -16.4 | <0.001 | 32.11 | -8.93 | <0.001 | 16.32 | 0.85 | 0.40 | -0.81 | -1.45 | 0.46 | -0.85 |
|  |  |  |  |  |  |  |  |  |  |  |  |  |  |  |  |  |  |
|  | **Task Stop-S** | *ANOVA* | 102.9 | <0.001 | 47.60 | 185.6 | <0.001 | 78.08 | 86.43 | <0.001 | 43.05 | 18.86 | <0.001 | 12.96 | 2.50 | 0.09 | -0.41 |
|  |  | *Post-hoc tests* |  |  |  |  |  |  |  |  |  |  |  |  |  |  |  |
|  |  | Control *vs* Otolith | 8.10 | <0.001 | 18.49 | -6.34 | <0.001 | 21.41 | -6.10 | <0.001 | 32.02 | 4.19 | <0.001 | 4.29 | 1.95 | 0.17 | -0.04 |
|  |  | Control *vs* Canal | 14.30 | <0.001 | 32.57 | -18.9 | <0.001 | 36.01 | -13.14 | <0.001 | 11.21 | -1.80 | 0.08 | -0.66 | 0.03 | 0.98 | -1.75 |
|  |  | Otolith *vs* Canal | 6.20 | <0.001 | 8.92 | -12.6 | <0.001 | 24.13 | -7.04 | <0.001 | 10.96 | -6.00 | <0.001 | 17.52 | -1.92 | 0.17 | -0.42 |
|  |  |  |  |  |  |  |  |  |  |  |  |  |  |  |  |  |  |
| Effect of task | **Condition Control** | *t-test*  *Stop-C vs Stop-S* | -8.60 | <0.001 | 17.61 | 0.31 | 0.76 | -1.70 | -4.00 | <0.001 | 4.53 | 1.68 | 0.10 | -0.47 | -4.38 | <0.001 | 5.57 |
|  | **Condition Otolith** |  | 4.10 | <0.001 | 4.81 | -16.6 | <0.001 | 36.01 | -8.34 | <0.001 | 16.90 | 3.81 | <0.001 | 4.05 | -1.28 | 0.21 | -0.99 |
|  | **Condition Canal** |  | 6.42 | <0.001 | 11.41 | -3.39 | <0.01 | 2.97 | -10.2 | <0.001 | 21.83 | -5.00 | <0.001 | 7.31 | -2.31 | <0.05 | 0.60 |
|  |  |  |  |  |  |  |  |  |  |  |  |  |  |  |  |  |  |

Task Stop-C: classical stop-signal task; Task Stop-S: sensorial stop-signal task; µ_Stop_: mean of the Gaussian component of the SSRTs distribution; σ_Stop_: standard deviation of the Gaussian component of the SSRTs distribution; τ_Stop_: mean of the exponential component of the SSRTs distribution; TF: trigger failure (percentage of stop-signal missed by the subject); GF: Go failure (percentage of Go stimuli missed by the subject); *Stat*: statistic; *p*: p-value; *BF*: log scale of Bayes factor BF_10_

**Supplementary Table 3** - **Condition and task effects on inhibition performance according to standard measures.** Standard statistics and Bayesian equivalents inform the effects of condition (upper part) and task (lower part) on standard measures of inhibition.

|  |  |  | Standard measures of inhibition | | | | | | | | |
| --- | --- | --- | --- | --- | --- | --- | --- | --- | --- | --- | --- |
|  |  |  | **SSRT** | | | **SSD** | | | **P_Inhib_** | | |
|  |  |  | *Stat* | *p* | *BF* | *Stat* | *p* | *BF* | *Stat* | *p* | *BF* |
| Effect of condition | **Task Stop-C** | *ANOVA* | 0.79 | 0.46 | -1.86 | 1.01 | 0.37 | -1.69 | 2.50 | 0.09 | -0.43 |
|  |  | *Post-hoc tests* |  |  |  |  |  |  |  |  |  |
|  |  | Control *vs* Otolith | -0.36 | 0.79 | -1.69 | 1.42 | 0.48 | -0.95 | -1.66 | 0.20 | -0.55 |
|  |  | Control *vs* Canal | -1.22 | 0.67 | -0.95 | 0.84 | 0.80 | -1.45 | -2.13 | 0.11 | -0.09 |
|  |  | Otolith *vs* Canal | -0.86 | 0.79 | -1.43 | -0.57 | 0.80 | -1.53 | -0.47 | 0.64 | -1.60 |
|  |  |  |  |  |  |  |  |  |  |  |  |
|  | **Task Stop-S** | *ANOVA* | 0.53 | 0.59 | -2.07 | 2.45 | 0.09 | -0.53 | 0.56 | 0.58 | -2.04 |
|  |  | *Post-hoc tests* |  |  |  |  |  |  |  |  |  |
|  |  | Control *vs* Otolith | 0.27 | 0.97 | -1.70 | 1.51 | 0.27 | -0.45 | -0.95 | 1.00 | -1.32 |
|  |  | Control *vs* Canal | -0.72 | 0.97 | -1.49 | 2.16 | 0.10 | 0.16 | -0.07 | 1.00 | -1.74 |
|  |  | Otolith *vs* Canal | -0.99 | 0.97 | -1.38 | 0.64 | 0.52 | -1.57 | 0.88 | 1.00 | -1.41 |
|  |  |  |  |  |  |  |  |  |  |  |  |
| Effect of task | **Condition Control** | *t-test*  *Stop-C vs Stop-S* | -1.92 | 0.06 | -0.09 | 6.88 | <0.001 | 12.73 | 3.26 | <0.01 | 2.67 |
|  | **Condition Otolith** |  | -0.99 | 0.33 | -1.29 | 5.00 | <0.001 | 7.29 | 2.52 | <0.05 | 1.01 |
|  | **Condition Canal** |  | -1.48 | 0.15 | -0.75 | 6.46 | <0.001 | 11.52 | 4.04 | <0.001 | 4.65 |
|  |  |  |  |  |  |  |  |  |  |  |  |

Task Stop-C: classical stop-signal task; Task Stop-S: sensorial stop-signal task; P_Inhib_: inhibition accuracy (percentage of Stop-trials with successful inhibition); SSRT: stop signal reaction time; SSD: stop signal delay; *Stat*: statistic; *p*: p-value; *BF*: log scale of Bayes factor BF_10_

**Supplementary Table 4 - Condition and task effects on decision-making according to standard measures.** Standard statistics and Bayesian equivalents inform the effects of condition (upper part) and task (lower part) on standard measures of decision-making performance.

|  |  |  | Standard measures of decision-making performance | | | | | | | | | | | | | | |
| --- | --- | --- | --- | --- | --- | --- | --- | --- | --- | --- | --- | --- | --- | --- | --- | --- | --- |
|  |  |  | **P_Go_** | | | **RT_Tot_** | | | **RT_Correct_** | | | **RT_Error_** | | | **IIV RT** | | |
|  |  |  | *Stat* | *p* | *BF* | *Stat* | *p* | *BF* | *Stat* | *p* | *BF* | *Stat* | *p* | *BF* | *Stat* | *p* | *BF* |
| Effect of condition | **Task Stop-C** | *ANOVA* | 1.24 | 0.29 | -1.50 | 3.20 | <0.05 | 0.05 | 2.88 | 0.06 | -0.20 | 1.26 | 0.31 | -0.64 | 3.11 | <0.05 | 0.28 |
|  |  | *Post-hoc tests* |  |  |  |  |  |  |  |  |  |  |  |  |  |  |  |
|  |  | Control *vs* Otolith | 0.12 | 0.91 | -1.74 | 0.49 | 0.62 | -1.32 | 0.46 | 0.65 | -1.64 | 0.49 | 0.63 | -0.98 | 1.12 | 0.35 | 0.60 |
|  |  | Control *vs* Canal | 1.42 | 0.48 | -0.13 | -1.90 | 0.12 | -0.15 | -1.81 | 0.15 | -0.31 | -1.06 | 0.61 | -0.60 | -1.37 | 0.35 | -1.11 |
|  |  | Otolith *vs* Canal | 1.30 | 0.48 | -1.24 | -2.40 | 0.06 | 0.70 | -2.27 | 0.08 | 0.52 | -1.55 | 0.43 | -0.35 | -2.49 | <0.05 | 0.22 |
|  |  |  |  |  |  |  |  |  |  |  |  |  |  |  |  |  |  |
|  | **Task Stop-S** | *ANOVA* | 0.24 | 0.79 | -2.29 | 0.55 | 0.58 | -2.05 | 0.41 | 0.67 | -2.19 | 1.74 | 0.19 | -0.80 | 2.36 | 0.10 | -0.55 |
|  |  | *Post-hoc tests* |  |  |  |  |  |  |  |  |  |  |  |  |  |  |  |
|  |  | Control *vs* Otolith | 0.64 | 1.00 | -1.60 | 0.95 | 1.00 | -1.08 | 0.84 | 1.00 | -1.15 | 1.87 | 0.20 | -0.16 | 0.01 | 1.00 | -1.75 |
|  |  | Control *vs* Canal | 0.10 | 1.00 | -1.74 | 0.08 | 1.00 | -1.74 | 0.14 | 1.00 | -1.74 | 0.95 | 0.70 | -1.07 | -1.88 | 0.19 | -0.13 |
|  |  | Otolith *vs* Canal | 0.54 | 1.00 | -1.62 | -0.87 | 1.00 | -1.39 | -0.70 | 1.00 | -1.51 | -0.92 | 0.70 | -1.23 | -1.89 | 0.19 | -0.64 |
|  |  |  |  |  |  |  |  |  |  |  |  |  |  |  |  |  |  |
| Effect of task | **Condition Control** | *t-test*  *Stop-C vs Stop-S* | 5.87 | <0.001 | 9.79 | 3.35 | <0.01 | 2.88 | 4.25 | <0.001 | 5.20 | -5.38 | <0.001 | 6.87 | -6.46 | <0.001 | 11.51 |
|  | **Condition Otolith** |  | 4.40 | <0.001 | 5.61 | 2.57 | <0.05 | 1.12 | 3.32 | <0.01 | 2.79 | -1.45 | 0.18 | -0.33 | -8.01 | <0.001 | 15.98 |
|  | **Condition Canal** |  | 4.99 | <0.001 | 7.28 | 3.37 | <0.01 | 2.91 | 4.00 | <0.001 | 4.53 | -0.02 | 0.99 | 1.46 | -2.99 | <0.01 | 2.03 |
|  |  |  |  |  |  |  |  |  |  |  |  |  |  |  |  |  |  |

Task Stop-C: classical stop-signal task; Task Stop-S: sensorial stop-signal task; P_Go_: decision-making accuracy (percentage of Go-trials with correct responses); RT_Tot_: mean reaction time for all Go-trials; RT_Correct_: mean reaction time for Go-trials with correct responses; RT_Error_: mean reaction time for Go-trials with errors; IIV RT: intra-individual variability in reaction times; *Stat*: statistic; *p*: p-value; *BF*: log scale of Bayes factor BF_10_

**Supplementary Table 5 - Decision-making performance in the task Stop-S as a function of the visual angle of stimulus.** Descriptive statistics (mean ± standard deviation) of standard measures informing the decision-making process as a function of the visual angle of stimulus from the subject’s 0°.

|  | Condition Control | | | Condition Otolith | | | Condition Canal | | |
| --- | --- | --- | --- | --- | --- | --- | --- | --- | --- |
|  | *Visual angle of stimulus* | | | *Visual angle of stimulus* | | | *Visual angle of stimulus* | | |
| Variable (unit) | *1°* | *2°* | *3°* | *1°* | *2°* | *3°* | *1°* | *2°* | *3°* |
|  |  |  |  |  |  |  |  |  |  |
| P_Go_ (%) | 78 ± 16 | 94 ± 6 | 98 ± 4 | 76 ± 22 | 93 ± 12 | 98 ± 6 | 76 ± 19 | 95 ± 9 | 98 ± 5 |
| RT_Tot_ (ms) | 457 ± 98 | 410 ± 99 | 384 ± 97 | 460 ± 119 | 394 ± 119 | 372 ± 104 | 459 ± 115 | 411 ± 116 | 377 ± 130 |
| RT_Correct_ (ms) | 450 ± 107 | 406 ± 100 | 383 ± 96 | 460 ± 120 | 386 ± 111 | 371 ± 105 | 454 ± 116 | 404 ± 115 | 374 ± 129 |
| RT_Error_ (ms) | 520 ± 131 | 521 ± 146 | 514 ± 77 | 446 ± 183 | 525 ± 261 | 511 ± 116 | 469 ± 173 | 583 ± 121 | 730 ± 260 |
| IIV RT (ms) | 151 ± 39 | 130 ± 38 | 113 ± 29 | 148 ± 54 | 124 ± 43 | 114 ± 32 | 157 ± 70 | 137 ± 71 | 137 ± 73 |
|  |  |  |  |  |  |  |  |  |  |

P_Go_: decision-making accuracy (percentage of Go-trials with correct responses); RT_Tot_: mean reaction time for all Go-trials; ms: milliseconds; RT_Correct_: mean reaction time for Go-trials with correct responses; RT_Error_: mean reaction time for Go-trials with errors; IIV RT: intra-individual variability in reaction times

SSRT: stop signal reaction time; ms: milliseconds; SSD: stop signal delay; µ_Stop_: mean of the Gaussian component of the SSRTs distribution; σ_Stop_: standard deviation of the Gaussian component of the SSRTs distribution; τ_Stop_: mean of the exponential component of the SSRTs distribution; GF: Go failure (percentage of Go stimuli missed by the subject); TF: trigger failure (percentage of stop-signal missed by the subject)

**Supplementary Table 6 - Effect of the visual angle of stimulus (task Stop-S) on correct reaction times.** Standard statistics and Bayesian equivalents inform the effect of the visual angle of stimulus on reaction times of correct trials.

|  | Condition Control | | | Condition Otolith | | | Condition Canal | | |
| --- | --- | --- | --- | --- | --- | --- | --- | --- | --- |
|  | *Stat* | *p* | *BF* | *Stat* | *p* | *BF* | *Stat* | *p* | *BF* |
| *ANOVA* | 46.94 | <0.001 | 24.95 | 34.48 | <0.001 | 19.35 | 22.08 | <0.001 | 12.80 |
| *Post-hoc tests* |  |  |  |  |  |  |  |  |  |
| 1° *vs* 2° | 6.16 | <0.001 | 8.43 | 5.98 | <0.001 | 7.69 | 3.85 | <0.001 | 5.05 |
| 1° *vs* 3° | 9.56 | <0.001 | 18.06 | 7.98 | <0.001 | 17.88 | 6.62 | <0.001 | 9.46 |
| 2° *vs* 3° | 3.40 | <0.01 | 6.66 | 2.00 | <0.05 | 0.46 | 2.76 | <0.01 | 2.12 |
|  |  |  |  |  |  |  |  |  |  |

*Stat*: statistic; *p*: p-value; *BF*: log scale of Bayes factor BF_10_

**Supplementary Table 7** - **Effect of the nature of trial response on reaction times.** Standard statistics and Bayesian equivalents inform the effect of nature of trial response (correct vs error) on reaction times.

|  |  | Task Stop-C | | | Task Stop-S | | |
| --- | --- | --- | --- | --- | --- | --- | --- |
|  |  | *Stat* | *p* | *BF* | *Stat* | *p* | *BF* |
| Condition Control | *t-test*  *Correct vs Error* | 4.01 | <0.001 | 4.04 | -7.20 | <0.001 | 13.51 |
| Condition Otolith |  | 4.36 | <0.001 | 3.74 | -2.51 | <0.05 | 1.01 |
| Condition Canal |  | -0.21 | 0.84 | -1.48 | -3.74 | <0.001 | 3.79 |
|  |  |  |  |  |  |  |  |

Task Stop-C: classical stop-signal task; Task Stop-S: sensorial stop-signal task;

*Stat*: statistic; *p*: p-value; *BF*: log scale of Bayes factor BF_10_

**Supplementary Table 8 -** **Condition and task effects on decision-making according to computational parameters.** Standard statistics and Bayesian equivalents inform the effects of condition (upper part) and task (lower part) on four main parameters of diffusion decision model.

|  |  |  | Computational parameters of decision-making process | | | | | | | | | | | |
| --- | --- | --- | --- | --- | --- | --- | --- | --- | --- | --- | --- | --- | --- | --- |
|  |  |  | **Response caution (*a*)** | | | **Drift rate (*ν*)** | | | **Non-decision time (*t_0_*)** | | | **Starting point (*z*)** | | |
|  |  |  | *Stat* | *p* | *BF* | *Stat* | *p* | *BF* | *Stat* | *p* | *BF* | *Stat* | *p* | *BF* |
| Effect of condition | **Task Stop-C** | *ANOVA* | 1.56 | 0.22 | -1.20 | 8.68 | <0.001 | 4.22 | 1.89 | 0.16 | -0.98 | 0.09 | 0.91 | -2.42 |
|  |  | *Post-hoc tests* |  |  |  |  |  |  |  |  |  |  |  |  |
|  |  | Control *vs* Otolith | 1.72 | 0.27 | -0.71 | -2.83 | <0.05 | 2.92 | -1.64 | 0.26 | -0.76 | -0.07 | 1.00 | -1.74 |
|  |  | Control *vs* Canal | 1.21 | 0.46 | -1.04 | 1.23 | 0.22 | -1.18 | -1.73 | 0.26 | -0.14 | -0.40 | 1.00 | -1.67 |
|  |  | Otolith *vs* Canal | -0.51 | 0.61 | -1.58 | 4.06 | <0.001 | 4.52 | -0.09 | 0.93 | -1.74 | -0.37 | 1.00 | -1.70 |
|  |  |  |  |  |  |  |  |  |  |  |  |  |  |  |
|  | **Task Stop-S** | *ANOVA* | 4.42 | <0.05 | 1.00 | 1.65 | 0.20 | -1.18 | 0.24 | 0.78 | -0.55 | 0.89 | 0.42 | -1.74 |
|  |  | *Post-hoc tests* |  |  |  |  |  |  |  |  |  |  |  |  |
|  |  | Control *vs* Otolith | 2.86 | <0.05 | 2.03 | 1.74 | 0.26 | -0.33 | <0.001 | 1.00 | -1.75 | 0.82 | 0.83 | -1.51 |
|  |  | Control *vs* Canal | 0.72 | 0.47 | -1.54 | 0.41 | 0.68 | -1.66 | 0.61 | 1.00 | -1.60 | 1.32 | 0.58 | -1.00 |
|  |  | Otolith *vs* Canal | -2.14 | 0.07 | 0.43 | -1.33 | 0.38 | -1.00 | 0.61 | 1.00 | -1.56 | 0.50 | 0.83 | -1.54 |
|  |  |  |  |  |  |  |  |  |  |  |  |  |  |  |
| Effect of task | **Condition Control** | *t-test*  *Stop-C vs Stop-S* | 10.88 | <0.001 | 23.62 | 11.02 | <0.001 | 23.97 | 8.31 | <0.001 | 16.82 | -1.51 | 0.14 | -0.70 |
|  | **Condition Otolith** |  | 14.82 | <0.001 | 32.61 | 13.07 | <0.001 | 28.82 | 5.58 | <0.001 | 8.96 | -0.38 | 0.70 | -1.68 |
|  | **Condition Canal** |  | 10.60 | <0.001 | 22.91 | 12.66 | <0.001 | 27.89 | 7.25 | <0.001 | 13.80 | 0.36 | 0.72 | -1.68 |
|  |  |  |  |  |  |  |  |  |  |  |  |  |  |  |

Task Stop-C: classical stop-signal task; Task Stop-S: sensorial stop-signal task; *Stat*: statistic; *p*: p-value; *BF*: log scale of Bayes factor BF_10_;

**Supplementary references**

Jeffreys, H. (1961). *Theory of probability* (Clarendon Press Ed.).
